# Supplementary material for: Legionella effector Lpg1137 shuts down ER-mitochondria communication through cleavage of syntaxin 17
Source: Nat Commun. 2017 May 15;8:15406. doi: 10.1038/ncomms15406 (PMC5440676; doi:10.1038/ncomms15406)

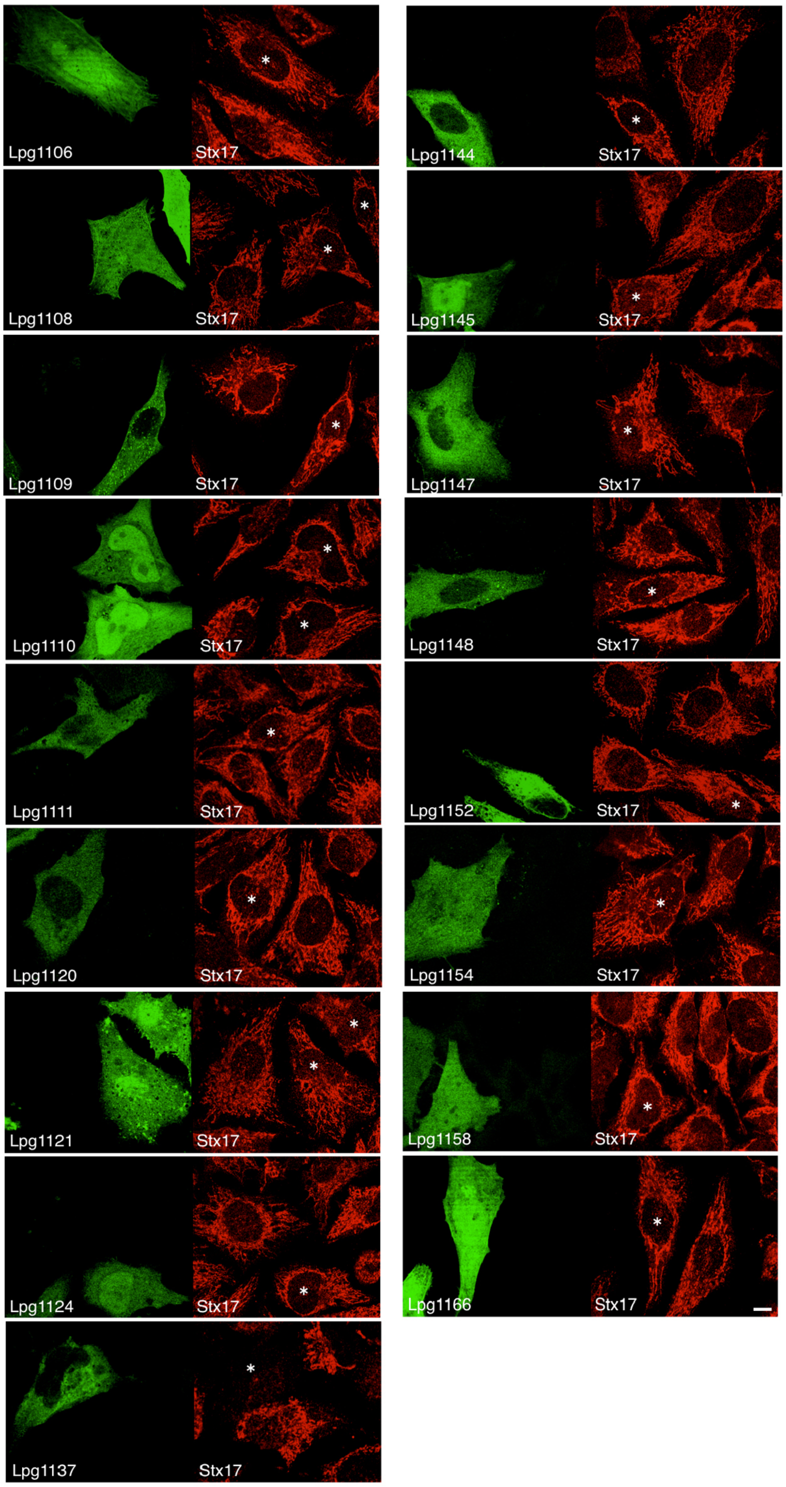


**Supplementary Figure 1 |** Effect of ectopic expression on Stx17 of effectors encoded by the genes in the island 2 locus. HeLa-FcγRII cells were transfected with one of the indicated 17 GFP-tagged constructs. At 24 h after transfection, cells were stained with an anti-Stx17 antibody. Asterisks indicate cells expressing GFP-tagged effectors. Scale bar, 5 μm.

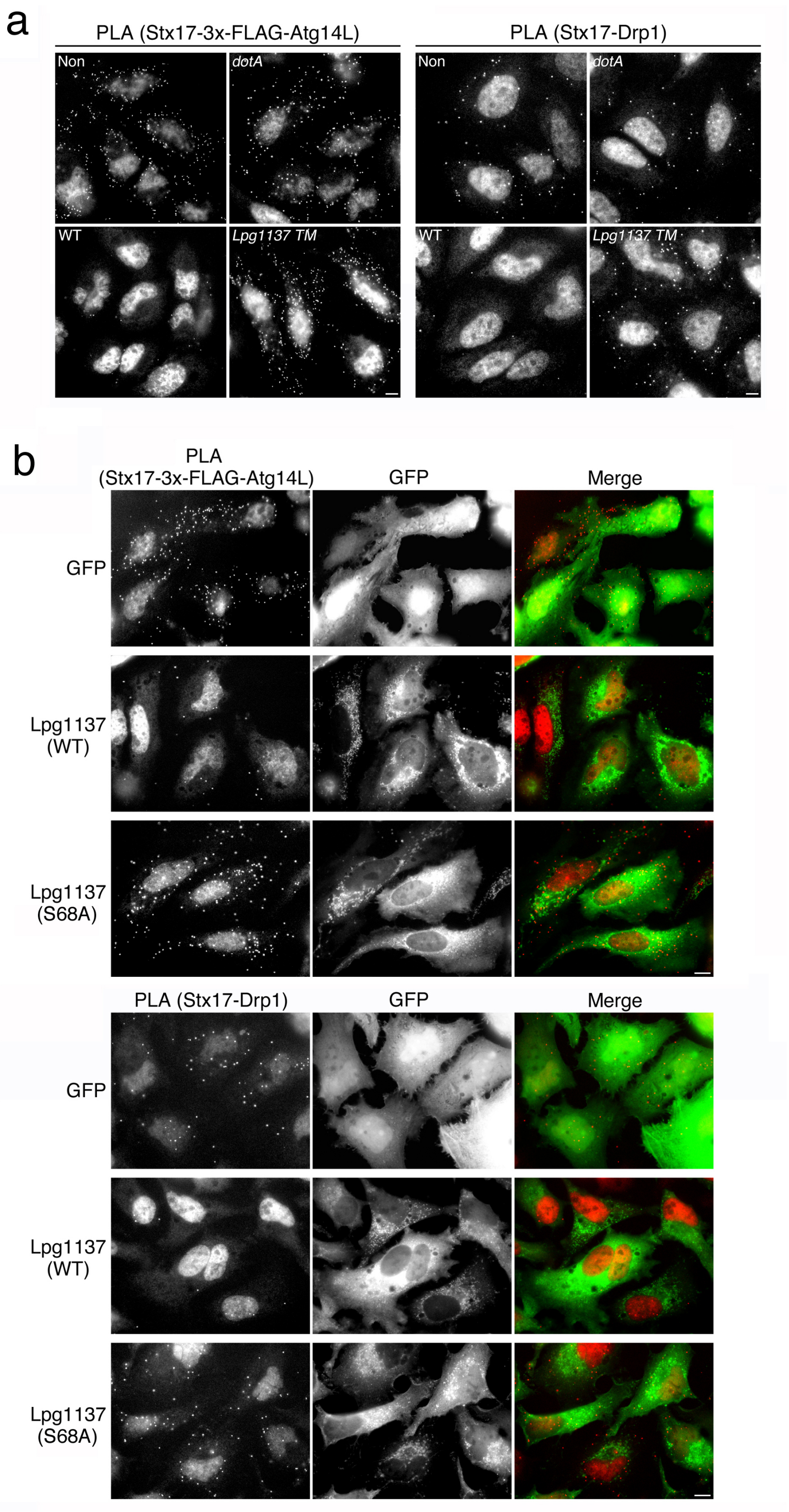


**Supplementary Figure 2 |** Lpg1137 subverts the interactions of Stx17 with Atg14L and Drp1. (**a**,**b**) HeLa-FcγRII cells were treated as described in the legend to Fig. 5. Scale bar, 5 μm.

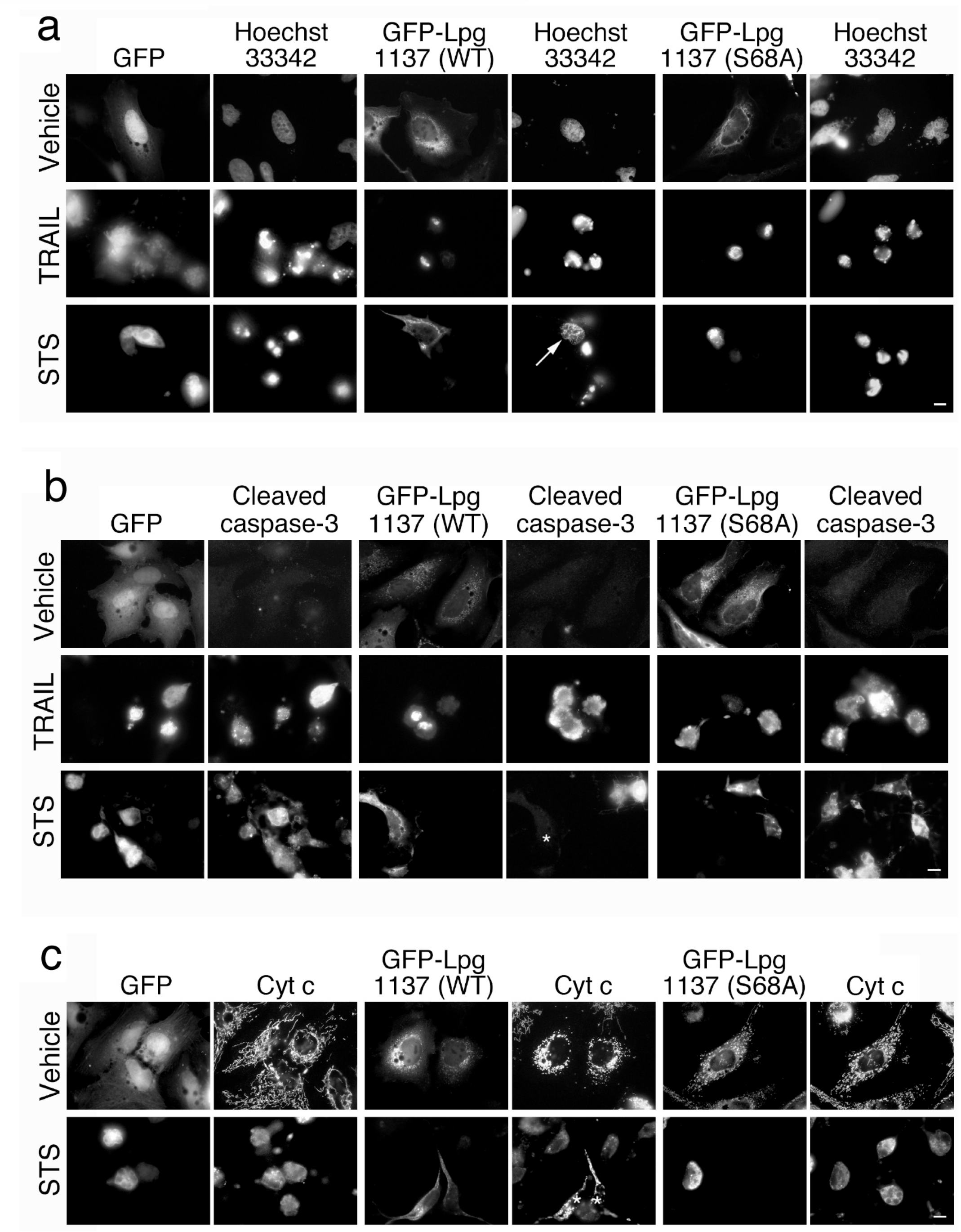


**Supplementary Figure 3 |** Ectopic expression of Lpg1137 blocks STS-induced apoptosis. HeLa-FcγRII cells were transfected with a plasmid encoding GFP, GFP-Lpg1137 wild-type or GFP-Lpg1137 S68A. At 24 h after transfection, cells were mock-treated (Vehicle) or treated with 500 ng/ml TRAIL or 1 μM STS for 4 h. After treatment, cells were fixed and stained with Hoechst 33342 (**a**), or an antibody against cleaved caspase-3 (**b**) or cytochrome c (**c**). Asterisks and an arrow indicate GFP-Lpg1137-expressing cells. Scale bar, 5 μm.

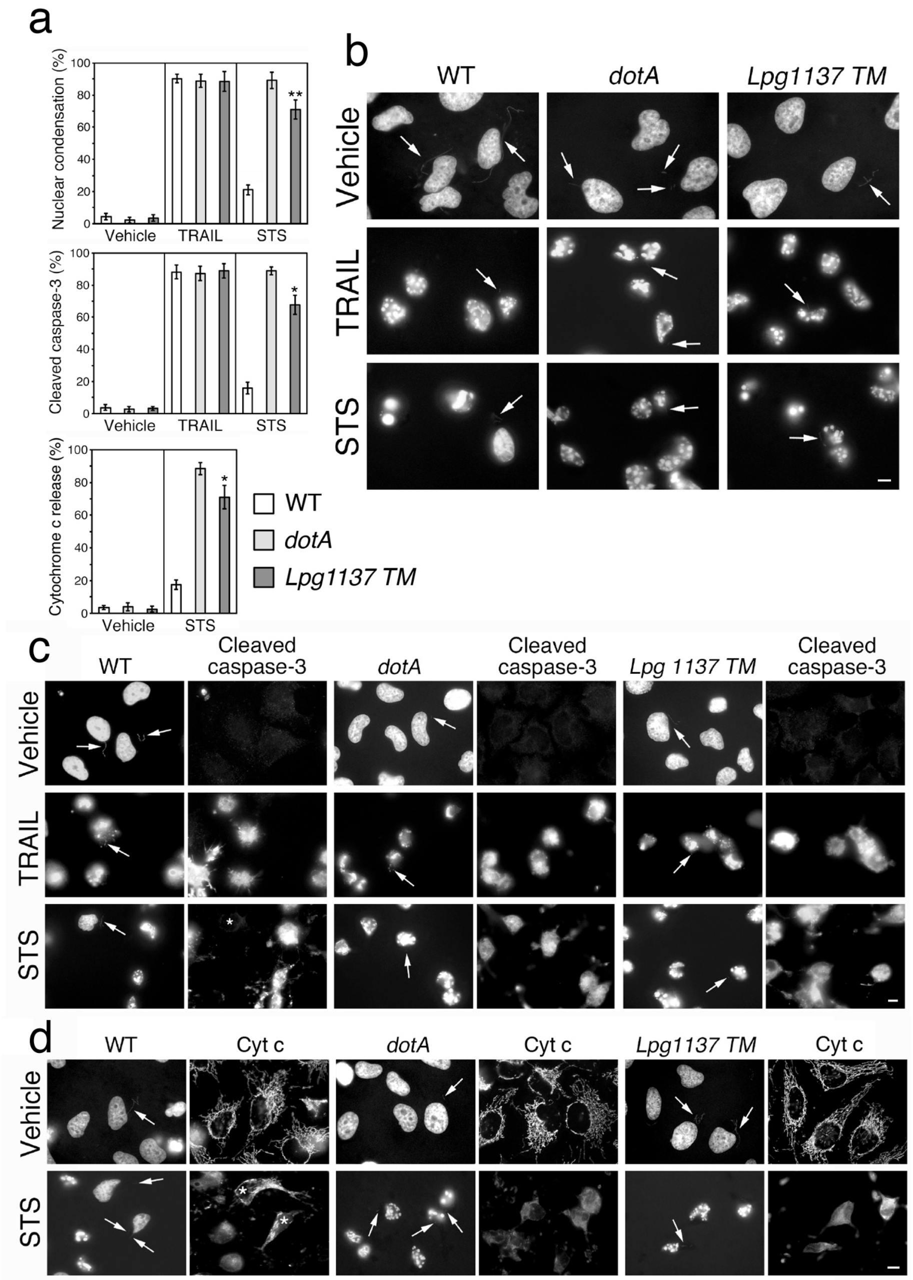


**Supplementary Figure 4 |** Infection of *Legionella* blocks STS-induced apoptosis. HeLa-FcγRII cells were infected with one of the indicated strains of *Legionella* at an MOI of 5 for 4 h. Cells were mock-treated (Vehicle) or treated with 500 ng/ml TRAIL or 1 μM STS for 4 h. After treatment, cells were fixed and stained with Hoechst 33342 alone (**b**), or with Hoechst 33342 and an antibody against cleaved caspase-3 (**c**) or cytochrome c (**d**). Scale bar, 5 μm. Quantification of the data is shown in (**a**). 100 infected cells were analyzed in each experiment. Values are means ± s.d. ($n$ = 4). *$P$ < 0.01 and **$P$ < 0.001 as compared with *Lpg1137 TM*. Arrows and asterisks indicate *Legionella*-infected cells.

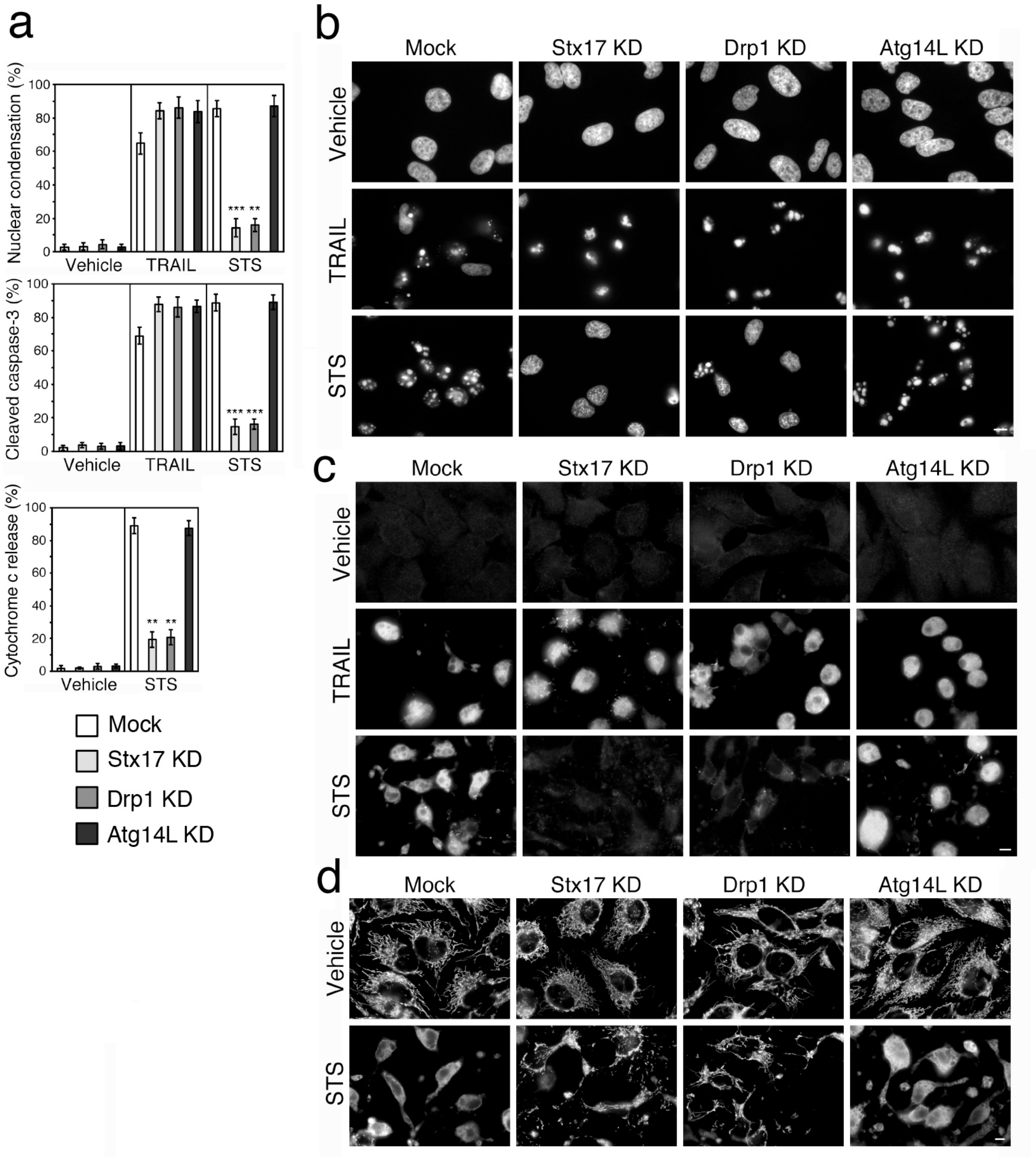


**Supplementary Figure 5 |** Silencing of Stx17 blocks STS-induced apoptosis. HeLa-FcγRII cells were transfected without (Mock), or with siRNA (KD) targeting Stx17, Drp1 or Atg14L. At 72 h after transfection, cells were mock-treated (Vehicle) or treated with 500 ng/ml TRAIL or 1 μM STS for 4 h. After treatment, cells were fixed and stained with Hoechst 33342 (**b**), or an antibody against cleaved caspase-3 (**c**) or cytochrome c (**d**). Scale bar, 5 μm. Quantification of the data is shown in (**a**). 100 cells were analyzed in each experiment. Values are means ± s.d. ($n$ = 4). $^{**}P < 0.001$ and $^{***}P < 0.0001$ as compared with Mock.

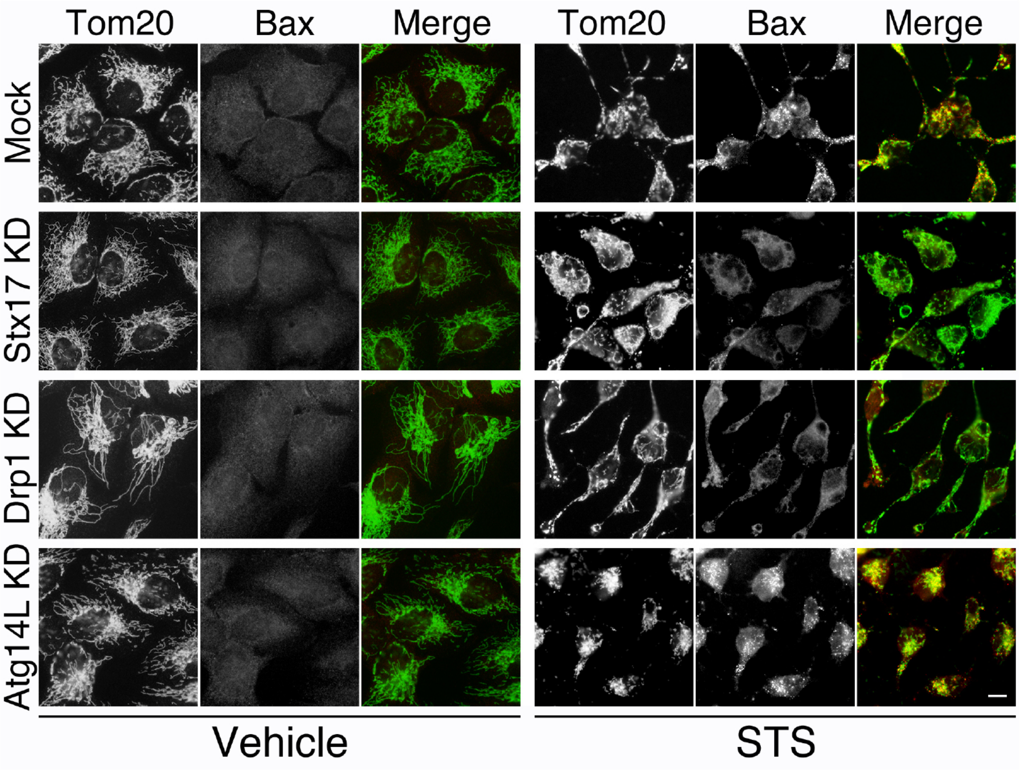


**Supplementary Figure 6 |** Silencing of Stx17 suppresses STS-induced translocation of Bax to mitochondria. HeLa-FcγRII cells were transfected without (Mock) or with siRNA (KD) targeting Stx17, Drp1 or Atg14L. At 72 h after transfection, cells were mock treated (Vehicle: left three columns) or treated with 1 μM STS (right three columns) for 4 h, fixed and then double stained with antibodies against Tom20 and Bax. Scale bar, 5 μm.

# Supplementary Figure 7

## Figure 1c

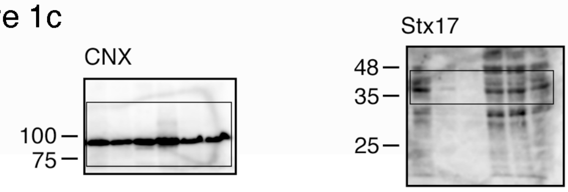


## Figure 1e

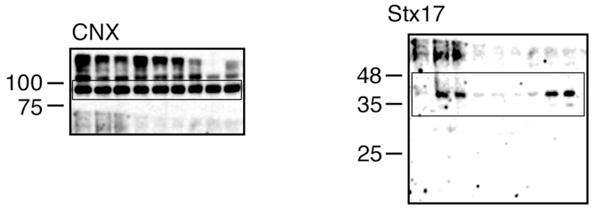


## Figure 2a

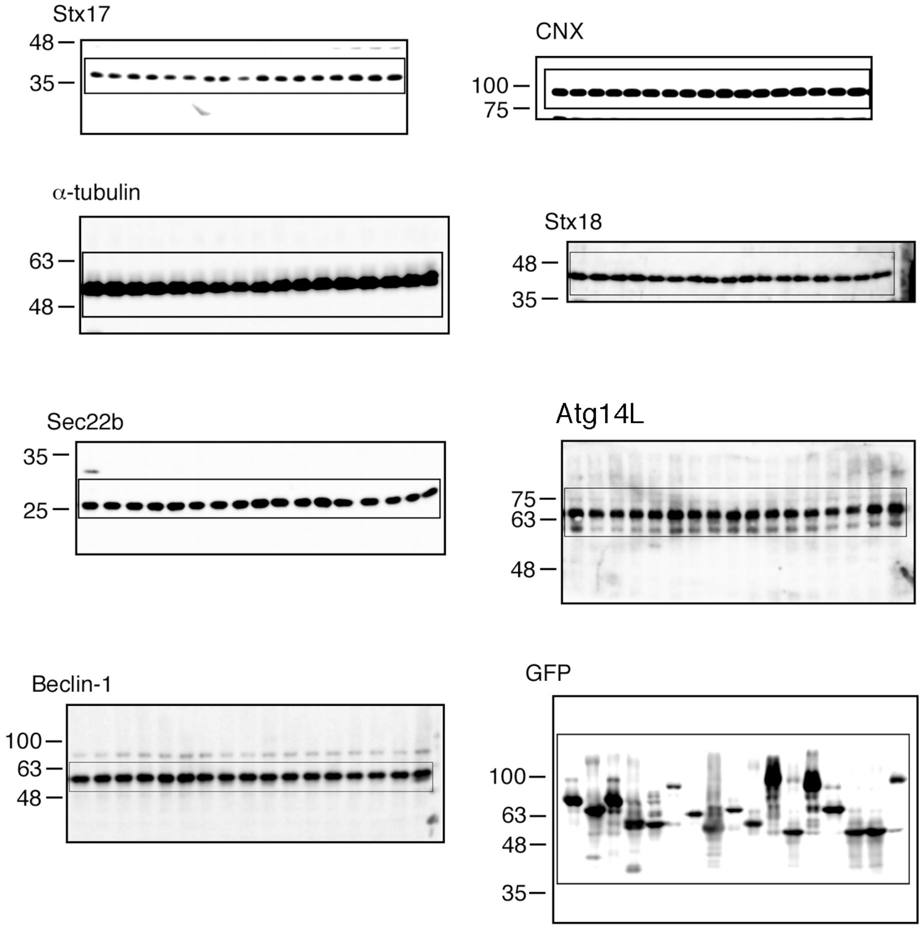


## Figure 2b

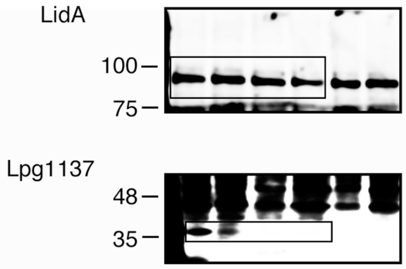


## Figure 2c

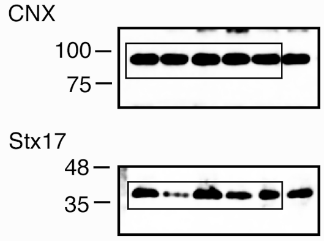

# Supplementary Figure 7 (continued)

## Figure 3a

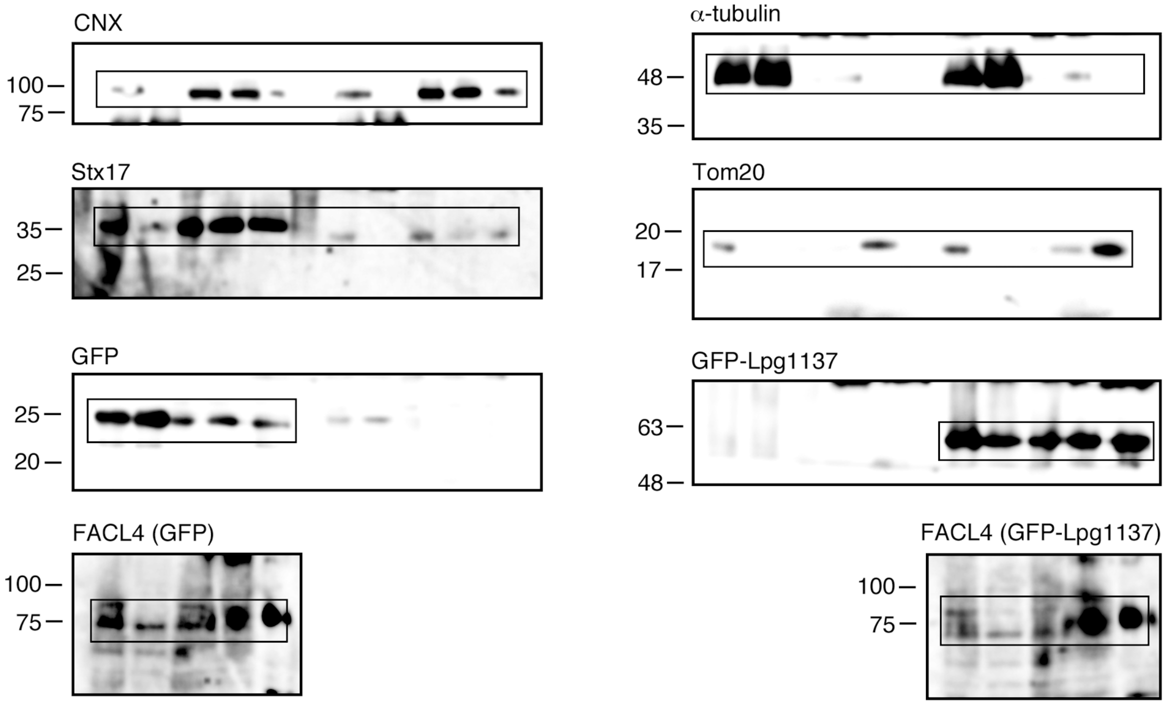


## Figure 3b

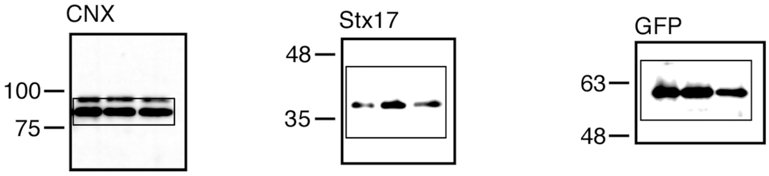


## Figure 3c

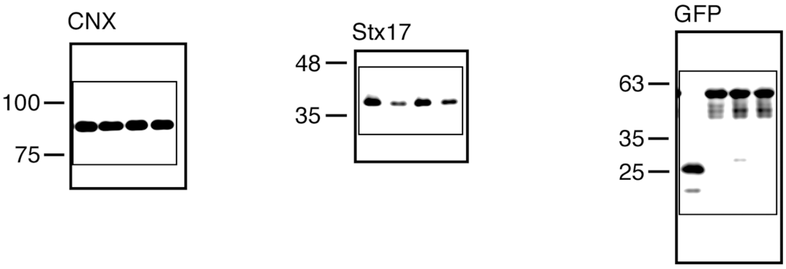


## Figure 3e

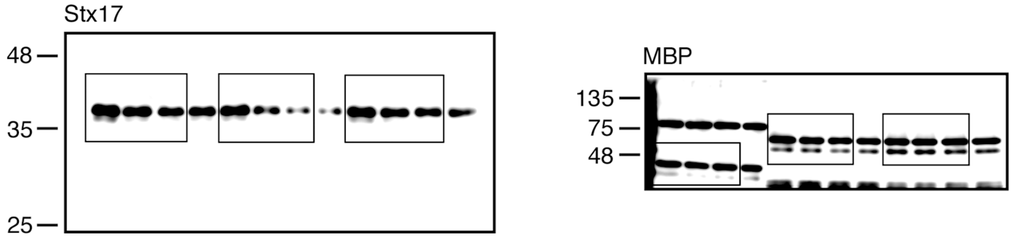

# Supplementary Figure 7 (continued)

## Figure 4a

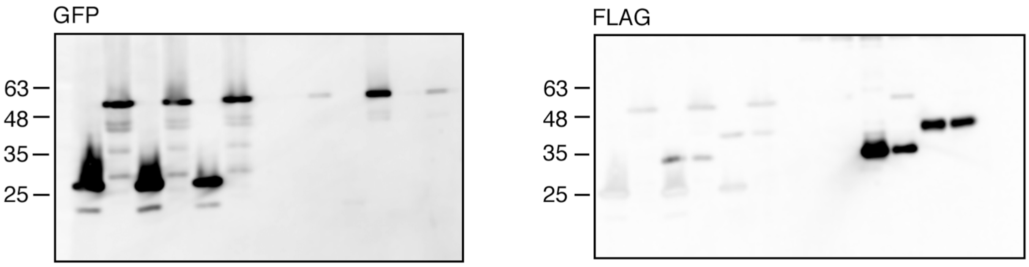


## Figure 4b

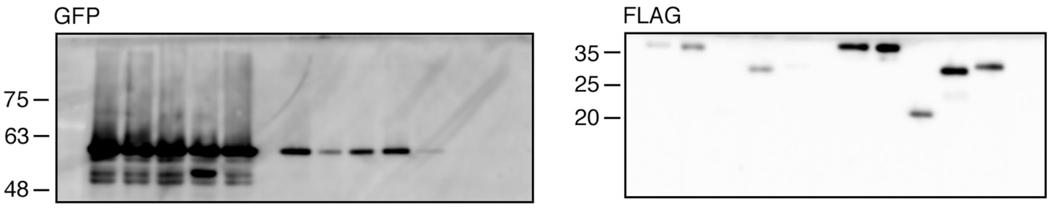


## Figure 4c

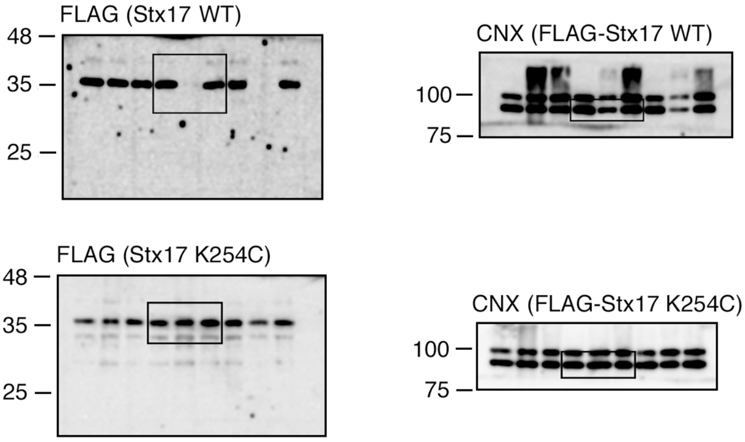


## Figure 6b

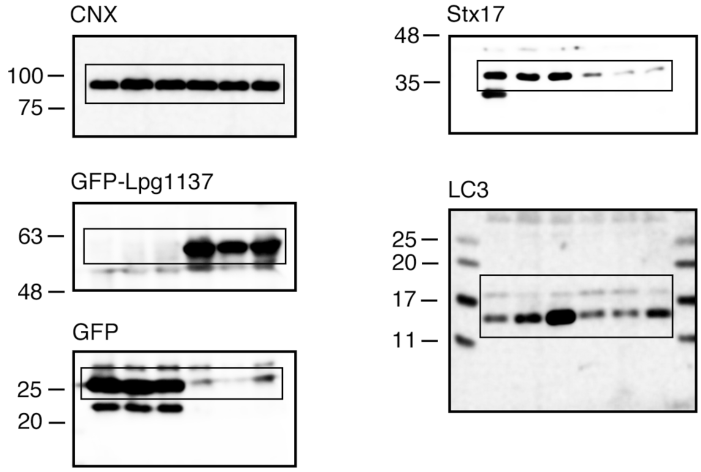

Supplement: Supplementary Information — Supplementary Figures [file ncomms15406-s1.pdf]
